# Ethical, Psychological and Social Un/certainties in the Face of Deemed Consent for Organ Donation in England

**DOI:** 10.1007/s10728-024-00492-0

**Published:** 2024-09-24

**Authors:** Laura L. Machin, Elizabeth Wrench, Jessie Cooper, Heather Dixon, Mark Wilkinson

**Affiliations:** 1 Lancaster University, Lancaster, UK; 2 City, University of London, London, UK; 3 Lancashire Teaching Hospitals NHS Foundation Trust, Fulwood, UK; 4 University Hospitals of Morecambe Bay NHS Foundation Trust, Kendal, UK; 5 Imperial College London, London, UK

**Keywords:** Deemed consent, Deceased organ donation, Donation values, Family veto, Opt-out, Uncertainty

## Abstract

Deemed consent legislation for deceased organ donation was introduced in England in 2020, and is considered a vital part of the new UK NHS Blood and Transplant’s 10-year strategy to increase consent for organ donation. Despite the legislation containing safeguards to protect the public, the introduction of deemed consent creates ethical, psychological and social un/certainties for healthcare professionals in their practice. In this paper, we offer insights into healthcare professionals’ perspectives on deemed consent, drawn from interview data with 24 healthcare professionals in an NHS Trust in England, prior to the introduction of the legislation. Whilst participants supported deemed consent in principle, they were concerned that it would present a threat to the nature of donation as a ‘gift’; the notion of informed consent (or non-consent); and the autonomy of donors, their relatives, and their own roles as health professionals, posing dilemmas for practice. We argue that healthcare professionals present themselves as guardians of potential (non)donors and thus as having ethics and integrity in their own practice. We draw conclusions around the values and principles that matter to healthcare professionals when contemplating consent in deceased donation which will be useful for organ donation committees and ethics forums.

## Introduction

In June 2021, UK NHS Blood and Transplant (NHSBT) launched a new 10-year strategy for organ donation and transplantation. A key focus of this strategy aims to increase consent for deceased organ donation by 2030, with the introduction of deemed consent for organ donation considered a vital component to meet this aim. Deemed consent for organ donation means people are assumed to have agreed to donate their organs after their death *unless* they register their decision to opt-out on the NHS organ donation register. Prior to this, people had to actively register their wish to donate their organs after their death, via the UKs Organ Donor Register (ODR). The deemed, or ‘opt-out’ legislation for organ donation was first introduced in Wales in 2015 [13], then England (May 2020) [25], and more recently Scotland (March 2021) [14] and Northern Ireland (June 2023) [24].

Embedded within the deemed consent policy are two safeguards thought to protect people’s ownership over their bodies. The first safeguard is the option for members of the public to ‘opt-out’ from the policy [28]. The idea being that an individual opposed to organ donation is more likely to opt-out under a system of deemed consent than someone who wishes to donate is to opt-in under an explicit consent system. This was observed when between five and six percent of the eligible population in Wales opted-out in the first three years of the deemed consent policy being implemented [23, 28, 30]. The second legislative safeguard is that family must be consulted and given the opportunity to provide formally recorded evidence that the individual would opt-out [26]. Overall ‘Family veto’, however, is still likely to be upheld by healthcare professionals regardless of evidence [23]. It is anticipated that fewer families will override the deceased’s wishes in a deemed consent system, and therefore a deceased person’s autonomy is perceived to be enhanced as a result [30].

Whilst the opt-out and family consultation are presented in the legislation as protecting the public, they are perceived by some bioethicists, clinicians and social scientists as creating ethical challenges for healthcare professionals that pivot around the rights of the dead to non-interference, self-determination and bodily autonomy [17, 30, 33]. These include the risk of healthcare professionals potentially violating people’s autonomy if patients have not formally registered their donation intensions in the event of their death [8, 28, 32]. As legal scholars and philosophers have highlighted, the deemed consent policy challenges our common understandings around consent—that consent is an active process, given explicitly [31, 33]. For healthcare professionals, therefore, this could mean proceeding with donation when there are doubts around a person’s donation intensions. As intensivists and bioethics have explained, deemed consent can result in patients who are indifferent to donation becoming donors by default [22], with their silence taken as tacit consent [20].

Moreover, bioethicists and social scientists have commented on the ethical uncertainty for healthcare professionals relating to the feasibility of overriding the family veto [23, 32]. This particularly applies if a deceased patient has not opted-out, which may lead to tensions between healthcare practitioners and relatives [1]. Some families may object because their loved ones had not formally registered their decision to opt-out, but had told their relatives whilst they were alive that they did not wish to donate [30]. For Farsides [10], this has the potential to create ethical uncertainty for healthcare professionals working on the frontline: “one of the biggest obstacles to successful organ donation is uncertainty. It needs to be known what people want to do or not to do” [10].

Finally, questions have been raised over how informed the public are when deciding to donate their organs, thereby making it challenging for healthcare professionals to trust the implied agreement within the deemed consent policy [28, 32]. We have written elsewhere regarding the troubling nature of aspects of organ donation and organ retrieval practices for those able to witness them, including relatives of deceased donors [19, 34]. Essentially, when relatives agree to donation, or the public do not opt-out, how knowledgeable are they on what happens before, during and after the removal of organs? For healthcare professionals then the ethical uncertainty exists over how informed the consent is, be it implicit (donor), or explicit (relatives). Yet, for Farsides [10], an autonomous person is not required to inform themselves more fully than they desire and donation should be viewed within the context of a person’s values. In practice then, healthcare professionals should act, according to Farsides [10], on what they gather to be the value-base of a patient rather than focusing on the understanding a patient may or may not have around organ retrieval practices.

Identifying areas of ethical uncertainty within a deemed consent system, such as those outlined above, is significant for developing our insight into the social, ethical and practical implications of the new deemed consent legislation for those working with potential (deceased) donors and their families. This paper offers insights into healthcare professionals’ perspectives on the deemed consent policy, in particular the opt-out and family veto aspects of the process, given the recent introduction of deemed consent to England and Scotland, and the new NHSBT organ donation strategy. To do this, we draw on interviews with clinicians and nurses in an NHS Trust in the North West of England, collected prior to the introduction of deemed consent, and as part of a project exploring the ethical dimensions of organ donation. The data we present focuses on the areas of un/certainty for healthcare professionals when discussing deemed consent for deceased organ donation. In what follows, we highlight that, whilst participants demonstrated their support in principle for a deemed consent system, they were concerned that it would present a threat to the nature of donation as a gift; the notion of informed consent (or non-consent); and the autonomy of donors, their relatives and their own roles as healthcare professionals, posing dilemmas for their practice. In doing so, we argue that healthcare professionals present themselves as guardians of potential (non)donors and thus as having ethics and integrity in their own practice. We draw conclusions around what we can learn about healthcare professionals’ ethical, psychological and social principles and values relating to deceased organ donation when considering an opt-out system.

### Methods

The study from which the data is drawn aimed to explore the dimensions of organ donation, with a particular focus upon the decision-making of healthcare professionals. We also wished to understand better how healthcare professionals involved in donation perceived the policies, processes, and practices surrounding organ donation, such as deemed consent. We discuss elsewhere other processes and practices such as the embedding of Special Nurses for Organ Donation (SNODs) in intensive care units, whose remit is to identify potential donors, gain consent from relatives and manage the donation process [11], and the reintroduction of donation after circulatory death (see [19]). A social constructionist approach [3] was adopted whereby the aim was not to unearth one true objective reality, but, instead, understand the ‘reality’ of organ donation as created through various meanings and practices ascribed to the process by healthcare professionals in the study [18].

The study took place at an NHS hospital Trust in North West England over the period December 2012 to April 2013 at a time when Wales had agreed to implement a deemed consent approach to deceased organ donation in 2015. Deemed consent was being considered for England during the time of the interviews. However, it is important for us to acknowledge the limits of our study in relation to the date of the interviews. We have explored participants’ perceptions and understandings of deemed consent rather than their experience of practicing within a deemed consent system. Participants were recruited from the Emergency Department, Intensive Care Unit and Operating Departments along with members of the Trust’s Organ Donation Committee as we considered these healthcare professionals to play a key role in organ donation within the Trust (Table 1). Recruitment of participants was made via an email invitation disseminated by departmental secretaries and participation was on a voluntary basis. Ethical approval was granted by Lancaster University in November 2012, and governance approvals were gained from the Trust’s Research and Development Department.

**Table 1:** The roles of healthcare professionals and the number of interviewees

Role of healthcare professional	Number of interviewees
Specialist Nurse for Organ Donation	1
Clinical Lead for Organ Donation	1
Intensive Care Unit—Ward Manager	2
Intensive Care Unit—Nurse	4
Intensive Care Unit—Doctor	2
Intensive Care Unit and Operating Theatre—Doctor	4
Operating Department—Manager	2
Operating Department—Nurse	1
Operating Department—Doctor	1
Operating Department—Practitioner	1
Emergency Department—Doctor	2
Emergency Department—Nurse	1
End-of-Life/Palliative Care—Nurse	2

Twenty four in-depth, semi-structured interviews with clinicians and nurses were conducted between January and April 2013 by HD. Consent was taken face-to-face prior to each interview, and it was explained that participants could withdraw their consent at any point during, and up to two weeks following the interview. Interviews lasted between 30 and 60 min approximately and were recorded on an audio device. An active interview approach was adopted, which enabled the research team to draw on their background knowledge of organ donation when designing the study [12]. A semi-structured interview guide based on a literature search was used, which focused on healthcare professionals’ understandings of the issues surrounding organ donation, such as autonomy, bodily ownership, altruism and consent and although issues of an ethical nature were discussed, the interviews were not conducted specifically from an ethical perspective. In particular, participants were asked about family involvement in decision-making, informed consent, and deemed consent with an individual’s understanding of terms such as ‘ethics’ also clarified. The guide was used to direct the questioning, with additional open questions used to facilitate more in-depth responses and allow for investigation of new knowledge raised.

Interview recordings were transcribed by a professional transcriber, who completed a Confidentiality Agreement before gaining access to the recordings. Reflexive thematic analysis was used to group together underlying patterns of shared meaning within the data and produce actionable outcomes from the analysis. The transcribed data was read multiple times to allow for reflective immersion and coded using Nvivo software. The codes focused on the main research questions: the areas of un/certainty for healthcare professionals when discussing deemed consent for deceased organ donation, how these un/certainties are created and portrayed by healthcare professionals, how healthcare professionals portray themselves and others when discussing the un/certainties surrounding the deemed consent system, why might healthcare professionals create un/certainty surrounding the deemed consent system, and what do they gain and lose by doing so. Codes were then grouped into over-arching themes which were continually refined as analytical outputs to ensure meaning-based interpretation [4, 5]. Although, previous research and researcher experience likely acted as a lens for subjective analysis, themes were produced as a meaning based analytical output with continuous development of themes and new themes based on underlying interpretation of the data.

## Results

### Donation Decision-Making

Overall, healthcare professionals presented themselves as supportive of a deemed consent system in principle as they perceived it would increase the number of donors, and raise awareness of organ donation practices. Yet, their support for a deemed consent system was dependent upon the information available to society, which in turn influenced people’s decision to be a donor or not. For these participants, whether a person chose to donate or not was of lesser importance, over the decision being an informed one.

*I personally quite like the opt-out system…I think it will let you increase the number of possible donations that you’ve got. As long as the people understand. (A1—1 ICU Consultant Anaesthetist)*

*I wouldn’t have any ethical qualms at all about it becoming an opt-out system as long as people knew what they were buying into. (AB1—2 Anaesthesia Consultant)*

*I like [opt out]. That’s better I think. As long as people are aware, like everybody is aware that it is an opt-out system I don’t see an issue with it. (A2-4 ICU Nurse)*

Participants thus prioritised public knowledge and understanding of the realities of organ donation practices over the aim of generating more donors. They cast doubt over how informed the public would be on opt-out by suggesting informational needs to make a decision might not be met in a large number of cases, although deemed consent would be couched within the moral good of increasing donors, donations and transplants.

Well, that’s a tricky one isn’t it. And I haven’t really thought about that in detail. Do I agree with it? You see in principle I do agree with it. But one of the issues, and we had this the other day with somebody, is people are paranoid about eyes. They don’t want their eyes donated for whatever reason. And so if you apply that to the opt-out system or somebody hasn’t got round to opting out or they didn’t know about it and they had no next of kin, well, they’re going to have all their organs taken including their eyes, which a lot of people don’t like the idea of. (C1-1*—*ED Consultant)

It’s a good idea in principle but whether we could pull it off and it be ethically correct and subject to the rules being bent a little. (B2-1*—*Staff Nurse)

I think there probably needs to be a bit more understanding. But it’s how do you get that message out there? Because the problem is you just scare people. (AB1-4 ICU & Anaesthetics Consultant)

Participants also posed ethical uncertainties during interviews when discussing peoples’ decision-making around organ donation in a system of deemed consent. At times, the deemed consent policy was understood to reduce the responsibility on the individual to make a decision. Future donors were thus constructed as passive rather than active decision-makers under a deemed consent policy, and/or concerns were raised over how informed donors were when choosing to donate. During interviews, participants referred to the public’s health decision-making behaviour. In this context, people were posed as the source of the ethical uncertainty by not making a decision with regard to donation.

*Most people say they would happily donate their organs but the majority of them aren’t on the organ donor register. Because even if you’ve just got to log on to the internet you’ve got to do something. Whether that means we should have an opt-out I’m not sure. (AB-1 ICU Consultant Anaesthetist)*

*I’ve got some misgivings about that [opt-out] really. I think the general engagement of people with major health service decisions or decisions affecting their wellbeing is fairly low and I think to expect people to opt-out is never really going to hit home to those people who haven’t got time to think about it. I think a lot of people have got a kind of it’s not going to happen to me attitude and I’ll get round to that one day and never do. I think organ donation, you’re always going to have demand outstripping supply and I think there are other ways of going about it really. (AB1—3 Anaesthetic Consultant)*

*…none of us think we’re going to die tomorrow…So what you would end up with is potentially tricky situations for us in ITU in saying, well, there’s no opt-out so we’re going to take the organs… (AE1—1 Consultant in Intensive Care Medicine)*

The deemed consent policy was presented as having the potential to generate more donors and potentially fulfil its aim of creating additional donors to meet transplant demand, although participants subtly cast doubt over how ethical the means of doing this were. The participants’ concern over the ethics of a deemed consent policy was further reinforced by the sense of how it would create perceived dilemmas for healthcare professionals*—*“the tricky situations” mentioned above and below*—*suggesting that the policy was not straightforward.

*The problem is who do you ask and how many people get involved in the decision? How do you decide whose opinions are important? Because, yeah, there may be, say, a husband or a wife, there may be sons and daughters and then there may be daughter-in-laws and son-in-laws, there may be ex-wives. There may be all sorts of people come forward and everybody always feels they have an equal right to the information and have an equal right to give their opinion. ( AB1-4 ICU Consultant Anaesthetist)*

*And at what stage do you make the opt-out age? 16, 18? How many 18 year olds actually ever think about their own mortality? I certainly didn’t when I was that age. (A1-2 ICU Consultant Anaesthetist)*

*…people’s feelings change. You know what it’s like when you’re younger, you often just go with what your family tell you to do… (E2-1 SNOD)*

The healthcare professionals thus presented themselves as concerned over bodily autonomy when discussing people’s engagement with, and society as vulnerable in the face of, a deemed consent system i.e. having organs removed that they might not have chosen to do so if it was an opt-in system for organ donation. This enabled participants to highlight their compassion for (non)donors, as well as their desire to act with integrity when engaging with patients, their bodies, and organ donation practices.

*My interest is that person laying in the bed and the family. They are the priority. They’re the ones that we’re caring for. (E2-2 SNOD)*

*I don’t think it is right to withhold the information. Because what happens is when you’re in that situation and then your relative is being kept alive you’re very much within your rights to say why didn’t anybody tell me this is what it was going to be like? It just adds stress and hurt to what is already a horrible situation I’m sure. But I think you probably should tell them but I think it might be at the cost of some organs. (B4—2 Theatre Operational Manager)*

*I hope it just raises awareness and people can go away and make a bit more of an informed choice themselves…If everybody has got a nice awareness they can make a nice informed choice of whether it’s something they actually want to do or not. (B2—1 Theatre Staff Nurse)*

The data from the study highlighted that there was a desire for donors to be recruited through ‘ethical’ means, and to do otherwise meant harms could result for donors and their relatives. Providing insight into organ donation practices was depicted as empowering (non)donors in their decision-making and choices, and minimising harms for the relatives of the donors, although it was rarely explicitly considered who should provide this insight e.g. healthcare professionals, SNODs, government, or NHS Blood and Transplant. The healthcare professionals championed the public’s right to information on organ donation and valued an openness around the realities of organ retrieval practices. Equally, the opt-out system could only be considered ethical if people were appropriately informed.

### Donation: The Gift of Giving or State Ownership?

During interviews, consultants and nurses in particular discussed their uncertainties around how the deemed consent system would work in practice. They expressed concern that the system equated to a loss of consent whereby the notion of consent was understood as an explicit act, and in turn created a potential loss of societal trust in the donation system.

*…if it was a case of they’re a potential donor, we are taking their organs no matter what you say, this is national policy, that would be a disaster. I think you would lose public faith and public involvement very, very quickly. You could just see the headlines, ‘Doctors stole my husband’s organs’. That would be disastrous. (A1-2 ICU Consultant Anaesthetist)*

*I think just a blanket saying we’re going to take everybody’s organs unless you do something about it I think is quite a dangerous precedent to set and a slippery slope to go down. Because, you know, where do you draw the line? (AB1—3 Anaesthetic Consultant)*

*But I don’t know if it would work because the whole thing is that it’s the gift of life and it’s about somebody wanting to help somebody else not making them. (A2-3—ICU Clinical Leader)*

In the quotes above, the healthcare professionals pose significant negative consequences by using dramatic language and large-scale hypothetical examples. This demonstrates their concern about the impact of the deemed consent policy on the continuation of the donation system, and on public perceptions of both the medical profession and of organs belonging to the state rather than the individual. The healthcare professionals position themselves as more concerned for individuals than for the societal need for donors, and therefore were supportive of (non)donors and relatives. Implicit in the extracts above is a preference by the healthcare professionals to work collaboratively with relatives and (non)donors as evidenced by the consultants’ aversion to “taking their [patients’] organs no matter what you [relatives] say”. In turn, that participants perceived there to be a loss of (active) consent in a deemed consent system implicitly positions them as valuing an individuals decision, rather than operating by “national policy”. It appeared participants aspired towards an equalling of power in the donation system between the state, the healthcare professionals, and the relatives and (non)donors.

Participants’ desire to uphold people’s autonomy over their bodies in a donation system, as well as retain clinical autonomy over their practices was also apparent when they discussed their uncertainty over *how* people could opt-out (or not) from the deemed consent system.

*How you enable people to opt-out I think would be difficult. Because you are depending on, if it became much more proactive from the point of view of—I’m going to use slightly distasteful terms here—gathering organs, you’d have to be very careful as to how stringently you ensured that everybody did have a proper option to opt-out. If it was more along the lines of “this [deemed consent] is our way of having a discussion about it”. But if we were to go “these are our organs to do with what we want” you’d have to have a very, very strong opt-out network there to be able to enable people who wanted to opt-out could opt-out. (A1-2 ICU Consultant Anaesthetist)*

*You’ve got elderly people who don’t use the internet or are not aware of the opt-out version and you don’t want them coming into hospital and then being harvested and it was something that they really, really didn’t want. And also you’ve got to have some sort of failsafe than it just happening anyway and, oh, I didn’t realise they’d opted-out. It’s one of them isn’t it. It’s a good idea in principle but whether we could pull it off and it be ethically correct and subject to the rules being bent a little. (B2—1 Theatre Staff Nurse)*

In the above quote, the nurse draws on everyday examples and common stereotypes around the elderly to reinforce the idea of the public as potentially vulnerable in relation to the deemed consent system. As such, the system was perceived to inadvertently create inequalities in terms of opting-out, which is reinforced by the choice of language used to describe the retrieval of organs*—*“harvest”*—*and the need for people to be protected from the policy*—*“failsafe”. Participants aligned themselves with (non)donors by raising concerns over people’s abilities to opt-out. By doing so, participants presented themselves as valuing people being enabled to enact their decision around organ donation in the face of a deemed consent policy, and promoting choice with regards to people’s bodies. The healthcare professionals thus positioned themselves as guardians and protectors of (non)donors who they see as made vulnerable by the policy.

At times, the opt-out function was presented by participants as protecting patients from state failure i.e. organs being retrieved from people who did not wish to donate. Yet, doubt was created around this “failsafe” when participants referred to the process of opting-out and/or someone’s ability to opt-out or their potential (in)action in the system.

*I think if you felt strongly enough then you would opt-out. I think there would have to be really clear processes in making it very easy for people to be able to do that so it wouldn’t be something that somebody felt they couldn’t or wouldn’t be able to do. (A4—2 ICU Ward Manager)*

*In terms of consent, I think it would be difficult to know whether patients have genuinely consented for organ donation with an opt-out system. (AB1—1 Anaesthesia/ICU Consultant)*

The opt-out process was described by healthcare professionals as needing to be transparent and accessible to all*—*“really clear processes”*—*in order for fairness to permeate deemed consent. By articulating the need for transparent processes, participants implied that the system needed to ensure donation remained voluntary and that (non)consent was fully informed. In turn, participants posed a dilemma in as much as they might never know a (non)donor’s wish around how they wanted their bodies to be treated in death.

*But then would there be people who if they don’t opt-out but you know that they really wouldn’t want it, it’s very difficult. You could have had these conversations but they’ve just physically don’t…They might not know how to do it. It’s very tricky…There isn’t any sort of clear cut way that you’d guarantee to get everybody and to know what everybody’s true wishes were. (A2—1 ICU Sister)*

Participants used language that questioned the authenticity of a (non)donors’ (in)actions e.g. how they would know what someone’s “true wishes” were, and whether they had “genuinely consented”. The doubt constructed here about the authenticity of a person’s wishes also had the effect of making health professionals vulnerable, in that they might find themselves in a situation where they were unwittingly acting against a person’s (unreported and undocumented when living) wishes. Participants thereby portrayed themselves as having an active regard for another person’s welfare, demonstrating compassion for their patients.

While healthcare professionals in this study presented themselves as supportive of organ donation and deemed consent, they generated uncertainty around how it could be implemented, which positioned them as having little control over the policy in practice. In many ways, the participants attempted to be simultaneously in favour, and also distance themselves from the policy. It was depicted as something being imposed upon them and the public, and they attempted to undermine the policy by casting doubt over how “well thought through it is”. They positioned themselves as vulnerable as a consequence, since they were the ones who had to conduct their practice according to the ‘ill-considered’ policy. In essence, they created doubt over how ethical the deemed consent policy was, and, in turn, implied that their concerns were coming from an ethical standpoint, thereby positioning themselves as having integrity in their practices, and in particular their decision-making relating to organ donation.

### Deemed Consent: Effective?

Participants generated much uncertainty around how successful the deemed consent policy would be in practice once implemented. The family veto, societal culture surrounding death and body ownership, and cultural understandings around the act of donation were all depicted as hindering the potential success of the deemed consent policy.

*I can’t see how we’re ever going to have an opt-out system where the family can’t override it…we’ve always really had real family involvement in after death…I think it’s not just changing the way we gain organs it’s changing the way in which we think about death and whose body is it. And certainly in this country I think most people believe the body really pretty much belongs to the family once they’ve died and this is a shift towards it belonging to the state. (AB1—4 Consultant in Anaesthetics and Intensive Care)*

*I suspect the family will still have the right to refuse so we are no further advanced. Or you will have a battle between doctors and the family potentially. So you might increase organ donations but I think the feel for organ donation would change and it wouldn’t be seen as being the gift that maybe it’s seen as being now. (AE1—1 Consultant in Intensive Care Medicine)*

Participants accepted that the deemed consent policy might lead to an increase in the number of donations, but its possible success was undermined and seen to lack justification when they identified intangible ‘costs’ to the culture of donation e.g. altruism and voluntariness. Moreover, the family were presented as powerful and influential in organ donation decision-making. In one way, this power was justified when participants made references to societal perceptions of body ownership resting with relatives of the deceased. Yet, the power was also presented negatively e.g. as a possible cause for conflict between healthcare professionals and family. In both instances, the deemed consent policy was implied as having either negative consequences—“battles”, “state ownership”*—*or simply ineffective, and unwarranted in the face of the family veto.

Participants tended to discuss the family veto in the context of people who had not opted-out and were therefore deemed to have agreed to donate their organs.

*I think there would still be the same problem that even if we presumed that everyone was for donation would that change the relatives? Would we still ask the relatives? And if we would how does that make it any different from the opt-in system? (A2-3 ICU Sister)*

*So is it going to solve anything? …even if I don’t opt-out and you come to the relatives and say he’s not opted-out so we’re having these organs and they say no, no are you going to do that? You’re not going to do that are you. (A2—2 ICU Charge Nurse)*

The ability for families to be involved in the organ donation decision-making process was presented by some participants as creating an ethical ‘certainty’*—*on the one hand, the deemed consent system was portrayed as no different in practice than an opt-in system. In a sense, all the challenges that the family veto presented for an opt-in system could be applied and considered relevant to an opt-out system. On the other hand, this created an uncertainty for some participants as to why the deemed consent system should be implemented at all; this implicitly undermined the effectiveness of the proposed deemed consent system, whilst also portraying relatives in a powerful and influential position in the face of a national policy. Alongside the possibility of potential donors’ wishes not being adhered to by relatives, so too can it apply to clinicians, as their autonomy and judgement were also stifled by the family veto. It was apparent then that participants valued autonomy and were mindful of the power dynamics that existed within organ donation decision-making.

### Donation Guardians

When contemplating the deemed consent system and the family, there was a respect for the deceased being part of a wider community i.e. a family. However, it was apparent that participants perceived the deceased as individuals and their decisions should be upheld.

*Personally, I think they (relatives) should have an opinion but I think what you want as the actual donor should be paramount. And I know it’s very, very difficult to implement that…Are the relatives respecting what the patient wanted? So you can sign a DNR and they have to respect that…I think if you’ve expressed this is what I want when I die then it should be respected, as would the way you want your funeral and anything else you want doing. It should be the same sort of thing. (B2—1 Theatre Staff Nurse)*

Healthcare professionals could therefore be deemed as the guardians of the dying or deceased patient, and their role was to facilitate the deceased patient’s wishes to donate organs. As such, the questioning around the potential inclusion of family in organ donation decision-making enabled participants to portray themselves as having the ability to make fitting judgements, and a desire to make organ donation decisions without being unduly influenced by external factors. Yet, there was also empathy from participants towards the relatives and the circumstances they found themselves in, thereby enabling participants to present themselves as compassionate.

Participants reinforced their position by framing the decision to donate as a ‘rights’ issue and by using language such as ‘choice’ and ‘respect’. The strength of such language acted as implicit justification for the feelings of relatives being a lower priority compared to an individual patient’s bodily autonomy and the need for healthcare professionals to guard patients’ bodily autonomy.

*I think you’ve got to respect the wishes of that person. Yeah, you’ve got to take into consideration the feelings of the family but that person has made that decision for themselves. You’d be doing them a disservice to actually not fulfil what they wanted. It is their right to choose what they want to do. I mean, if they decided to put their body up for research, who are we to question? They’ve made that decision. The family can’t say no to that so why should they be able to say no to organ donation? (B4—1 Theatre Clinical Manager)*

Participants described alternative circumstances when a dying or deceased person’s decision was respected, and families could not overturn it, thereby generating doubt as to why it was permitted in the context of organ donation. Again, the theme of power emerged, with deceased patients vulnerable to the feelings and wishes of relatives.

*Opt-out, the problem is that when the family disagree you don’t know if was just their wishes or the patient’s wishes then. (AB1—4 Consultant in Anaesthetics and Intensive Care)*

The relatives were presented as the most powerful in the context of organ donation decision-making. In turn, the notion of the family veto as a safeguard in the introduction of the new deemed consent policy for deceased organ donation was therefore undermined by participants during interviews creating a position for healthcare professionals to protect their patients and guard their bodies in their death.

## Discussion

Understanding uncertainties around organ donation for healthcare professionals has been identified as crucial to secure the future of this system [10]. The findings of this paper show that, although organ donation and deemed consent overall were supported by healthcare professionals, there were numerous ethical uncertainties constructed by participants in relation to deemed consent for donation. These were particularly prominent when discussing the opt-out and the family veto elements of the deemed consent policy. In our study, the constructed ethical uncertainties stemmed from a range of sources particularly the people and processes involved in the deemed consent policy, such as public, relatives, non-donors, donors, and healthcare professionals, and opt-out process and organ donation decision-making.

These constructed uncertainties enabled aspects of the deemed consent policy to be called into question about whether it was needed or effective, and at what cost to the donation system. The effectiveness of ‘deemed consent’ implementation in 2020 is still difficult to assess due to it’s implementation in the COVID-19 pandemic where organ donation rates dropped significantly [26]. Prior to implementation, participants questioned the ethics and autonomy of such a policy. Participants questioned how informed the consent was in a deemed consent system, and cast doubt over whether potential donors really had consented or merely had not opted-out of the system for a range of reasons.

Consequently, a number of ‘vulnerabilities’ were brought to the fore. Non-donors were presented as vulnerable by the deemed consent policy: they may become a donor when they may have otherwise chosen not to donate under a different system, or vice versa if their family vetoed their donation when they had not opted-out. Another group perceived to be vulnerable by the deemed consent policy were the relatives of the deceased due to the policy diminishing their societal and cultural ownership over the deceased’s body. A final group made vulnerable were healthcare professionals who could find themselves acting against a patient’s (unknown) wishes if they had not formally registered their wish not to donate. In turn, it requires healthcare professionals to position themselves as ‘ethical’ which entailed acting with integrity, having compassion for others, making fitting judgements, and being trustworthy.

Taken collectively, our findings have highlighted participants’ concerns over state ownership and national policy dictating organ donation. Participants critiqued the deemed consent policy giving power to healthcare professionals to remove organs irrespective of objections from relatives. Members of the public in England and Scotland who self-reported to opt-out addressed similar concerns regarding a lack of autonomy and medical mistrust being the underlying reasons behind their opt-out decision [21]. Deemed consent implemented in Brazil in 1997 failed and this was thought to be in part due to a lack of trust in the healthcare system and an unwillingness of healthcare professionals to initiate organ donation without family consent [9]. Trust in the healthcare system and state ownership increasingly important concepts to consider in England after the UK Human Tissue Act 2004 was implemented as a response to the organ retention scandals which occurred due to medical paternalism and a lack of informed consent [2].

As deemed consent has been implemented in England since data collection, the findings were compared with healthcare professional perceptions post-implementation to understand whether initial concerns remained. The #options 2020 survey asked healthcare professionals their views on the recent implementation of deemed consent and raised similar concerns post-implementation. Loss of autonomy was highlighted as a main theme, with concerns remaining over informed consent and awareness of public to changes [7]. Therefore, ethical uncertainties identified in our study are arguably reflected in the way the deemed consent system has been implemented in England.

Participants argued for donation to be voluntary and repeatedly returned to the principle of bodily autonomy and ownership during interviews, similar to the rights of the deceased to non-interference and self-determination discussed by others elsewhere [17, 30, 33]. Thereby generating the need for guardians and champions for those vulnerable in a deceased organ donation system whilst also acting within a caring ethical climate [6]. Current guidance on deemed consent in England suggests donation should not proceed if contact cannot be made to a qualifying individual which could advocate for their preferences, allowing a layer of protection for the deceased [15, 27].

Furthermore, enabling people in their decision-making and promoting choice were deemed significant to participants when they considered the public’s awareness of the deemed consent policy and people’s abilities to opt-out. Ethical concerns post-implementation have been raised more recently about the awareness of the public to deemed consent [7, 26]. Implementation in England occurred during the COVID-19 pandemic where public awareness campaigns were likely to be overshadowed whereas Scotland delayed deemed consent implementation due to similar concerns [26]. Comparisons have been made specifically between the success of Spain’s deemed consent and the stagnation of donation in England after the implementation of deemed consent [29]. The processes in Spain are thought to be more streamlined and embedded within the healthcare system, and public education and clarity in the organ donation process are thought to be large players in its success [6, 29]. The Netherlands also implemented deemed consent during the COVID-19 pandemic but made it a legal requirement for an individual to register their preference on whether they would like to donate their organs [16]. It would therefore be interesting to understand the perceptions of healthcare professionals in the Netherlands to determine whether this legal requirements reduces some uncertainty in the decision making process [16].

Through these constructed uncertainties, it is possible to observe values and principles that matter to healthcare professionals involved in deceased organ donation, some of which have been identified by others such as autonomy [7, 8, 30]. Post-implementation, the #options 2020 survey highlighted that transplant centre employees still request additional guidance on family influence, information families should be provided with and the safeguards that are in place [7].

**Fig. 1:**
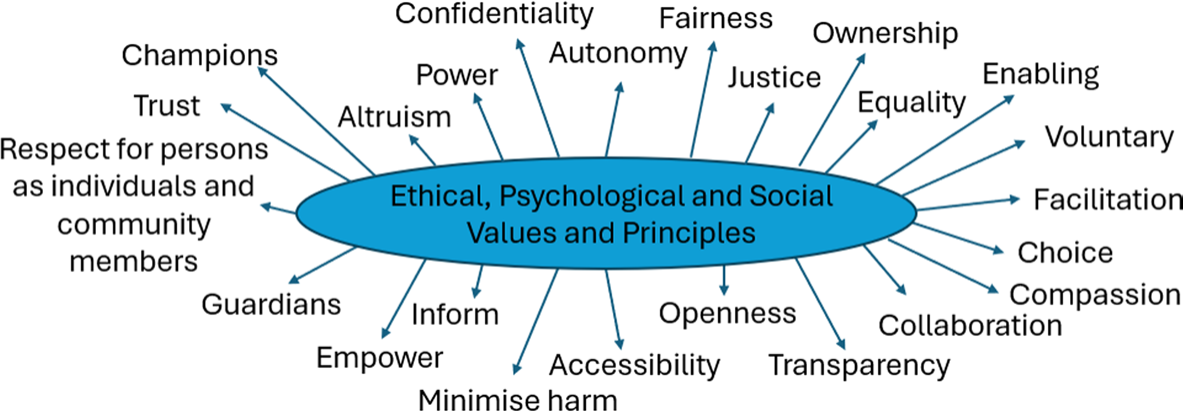
Overarching ethical, psychological and social values and principles surrounding deceased organ donation practices to provide direction and guidance of decision making, and can be tailored to specific institutions and government policy

When contemplated together therefore, these values and principles can provide guidance to support decision making surrounding deceased organ donation practices. With regards to consent specifically, these ethical, psychological and social values and principles could also act as a foundation to inform and influence, and incorporate donation more broadly (see Fig. 1).

The proposed ethical, psychological and social values and principles arguably capture what healthcare professionals constitute ethical practice in deceased organ donation with regards to consent specifically. It is what healthcare professionals consider morally acceptable when contemplating consent in deceased organ donation. Considering the implementation of deemed consent in England and research post-implementation suggesting these uncertainties still exist, providing additional guidance to healthcare professionals is increasingly important [7]. Having insight into these overarching values and principles and the un/certainties of healthcare professionals may be useful for organ donation committees within hospital trusts, or may hold relevance for the ethics forum proposed in the new NHSBT organ donation strategy. Further research is required to explore if such values and principles are personal and individualised to each healthcare professional, as we have discussed elsewhere when contemplating extending the remit of conscientious objection to some organ donation practices [19]. Alternatively, the values and principles proposed here may be generic and universal principles that apply to deceased organ donation such as those that can be identified for medical ethics or research ethics.

## Limitations

There are two main limitations of this study. The first is the date of data collection between December 2012 and April 2013, prior to the implementation of deemed consent within England in 2020. Even though the limitation remains, this data allows comparisons to be made between the opinions of healthcare professionals prior to implementation and the present landscape of deemed consent. Although further evidence needs to be collected on the current landscape, this data provides a useful insight into the uncertainties health care professionals experience and the ethical, psychological and social values and principles which can be considered by organ donation committees and healthcare professionals to reduce ethical uncertainty when making decisions about organ donation. The second limitation is the data was only obtained by one Trust. Findings could differ between Trusts, but the data still provides a guidance that is likely to be relevant to other trusts within England.
